# Magneto-Hybrid Nanofluids Flow via Mixed Convection past a Radiative Circular Cylinder

**DOI:** 10.1038/s41598-020-66918-6

**Published:** 2020-06-26

**Authors:** E. R. EL-Zahar, A. M. Rashad, W. Saad, L. F. Seddek

**Affiliations:** 1 Department of Mathematics, College of Science and Humanities in Al-Kharj, Prince Sattam bin Abdulaziz University, Al-Kharj, 11942 Saudi Arabia; 2 Department of Basic Engineering Science, Faculty of Engineering, Menoufia University, Shebin El-Kom, 32511 Egypt; 3 Departments of Mathematics, Aswan University, Faculty of Science, Aswan, 81528 Egypt; 4 Department of Engineering Mathematics and Physics, Faculty of Engineering, Zagazig University, Zagazig, 44519 Egypt

**Keywords:** Mechanical engineering, Materials science

## Abstract

The goal of the current analysis is to scrutinize the magneto-mixed convective flow of aqueous-based hybrid-nanofluid comprising Alumina and Copper nanoparticles across a horizontal circular cylinder with convective boundary condition. The energy equation is modelled by interpolating the non-linear radiation phenomenon with the assisting and opposing flows. The original equations describing the magneto-hybrid nanofluid motion and energy are converted into non-dimensional equations and solved numerically using a new hybrid linearization-Chebyshev spectral method (HLCSM). HLCSM is a high order spectral semi-analytical numerical method that results in an analytical solution in *η*-direction and thereby the solution is valid in overall the *η*-domain, not only at the grid points. The impacts of diverse parameters on the allied apportionment are inspected, and the fallouts are described graphically in the investigation. The physical quantities of interest containing the drag coefficient and the heat transfer rate are predestined versus fundamental parameters, and their outcomes are elucidated. It is witnessed that both drag coefficient and Nusselt number have greater magnitude for Cu-water followed by hybrid nanofluid and Al_2_O_3_-water. Moreover, the value of the drag coefficient declines versus the enlarged solid volume fraction. To emphasize the originality of the current analysis, the outcomes are compared with quoted works, and excellent accord is achieved in this consideration.

## Introduction

Recently, great interest and attention of numerous researches have been attracted towards nanofluids in order to improve the thermal conductivity of the fluid, heat transfer performance and fluid flow characteristics, etc. The nanofluid is described as a base fluid with suspended nanometer sized particles (diameter lower than 100 nm) from just one type of materials. These materials commonly are made of carbides (SiC), oxides (Al_2_O_3_, CuO), metals (Cu, Ag), and carbon nanotubes (CNTs, MWCNTs, diamond)^1^, while water, engine oil, glycol, etc. are widely can be applied as the base fluid. Most of nanofluid applications focus on all practical fields which require heat transfer or cooling or drug delivery such as nuclear reactor cooling^2^, automotive^3,4^, solar collectors^5,6^, refrigerators^7–9^, heat exchanger^10,11^, and electronics cooling^12–14^. Choi^15^ was the first to show the concept and benefits of employing the nanoparticles scattered in a primary fluid in order to promote the heat transport. Kumar *et al*.^16^ introduced a pattern for heat conduction in nanofluids flow. Several studies have been introduced by Das^17^, Mintsa^18^ and Zhu^19^ on the factors affected the thermal conductivity of nanofluid such as the stability, kind, size, shape and concentration of the dispersed nanoparticles, fluid temperature and kind of primary fluid applied.

In the resumption of nanofluids study, the investigators have also tried to apply hybrid nanofluid lately, which is engineered by suspending various nanoparticles either in composite or mixture form. In addition to creating a desired and great thermal conductivity, utilizing hybrid nanofluids can drive to the final cost depression and the convenient and acceptable stability of nanofluids and can provide the groundwork of the enormous manufacturing. The notion of empolying hybrid nanofluids is to further improve the heat transfer and pressure decline features by tradeoff between characteristics and disadvantages of individual suspension, attributed to perfect portion rate, better thermal network, and synergistic effect of nanomaterials (see^20^). Suresh *et al*.^21^ achieved a hybrid nanofluid flow of Cu-Al_2_O_3_/water with different volume concentrations. More recently, investigations on hybrid nanofluid flow by means of various physical conditions have been reported by^22–28^.

Mixed convection is one of the major fields for researches because of its importance to enhance thermal properties of the heat transfer. It also considers the general case of convection which occurs in several technological and industrial applications in nature, such as electronic devices cooling, drying technology, solar energy storage, float glass production and food processing. Pak and Choi^29^ have experimentally reported that the heat transfer convective using nanofluids in the turbulent flow regime. Syakila and Pop^30^ used a vertical flat plate to study mixed convection flow of nanofluids embedded in a porous medium. Many investigations on nanofluids flow by mixed convective can be found in^31–34^.

Lately, magnetohydrodynamic (MHD) has received noticeable consideration owing to its great area of applications particularly in: engineering, chemical technology, petroleum, environmental and geophysics sciences. MHD involves utilize of the magnetic field commonly used orthogonal to the fluid flow which has potential to generate a drag force famous as the Lorentz force. This force act versus the fluid flow which in turn impacts the velocity of the fluid in the essential trend. Waqas *et al*.^35^ studied the MHD mixed convection flow of non-Newtonian liquid over a nonlinear stretched sheet. Zhao *et al*.^36^ discussed the effect of magnetic field heat transfer of nanofluids in microchannels. Several other significant studies in this concern are due to^37–40^.

In all the afore investigations, scientists and scholars analyzed flow and heat transfer attributes of either nanofluids or hybrid nanofluids due to surface of solid geometry with various aspects. The persistence of present exploration is to deliver an arithmetical survey of MHD mixed convection flow of hybrid nanofluid around a radiative horizontal circular cylinder with thermal convective boundary conditions. Inspired by these facts, this investigation involves more possible applications in several engineering areas like as nuclear safety, transportation, manufacturing, military, pharmaceutical, naval structures, acoustics, microfluidics buildings or cooling of flush-mounted electronic heaters in modern electronic devices. The numerical solution of this problem is obtained using a new hybrid linearization–Chebyshev spectral method (HLCSM) is developed for obtaining the numerical solution of the considered problem. HLCSM is a high order specteral semi-analytical numerical method that results in analytical solution in η-direction, and so the solution is valid in overall the domain, not only at the grid points. A discussion of the plotted numerical results of the involved parameters versus associated distributions is presented.

## Problem Configuration

**Figure 1:**
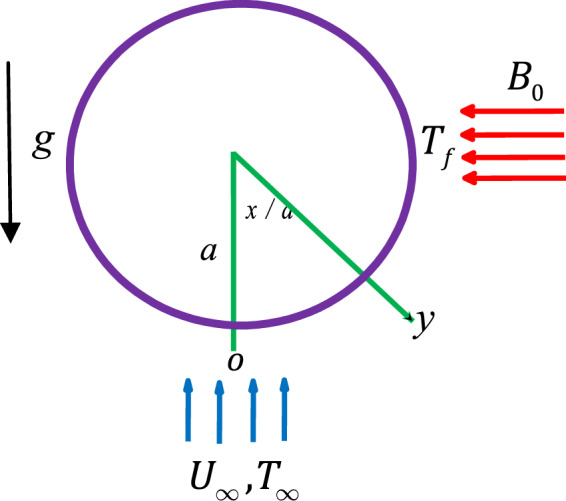
Schematic physical model.

In this segment, it is shown that the mixture of two different kinds of nanoparticles (hybrid nanofluid) disseminated in a base fluid, promotes the heat capacity of the base fluid. For example, Al_2_O_3_ shows more chemical inertness and stability, but exhibits weaker thermal conductivity with respect to metallic nanoparticles. Moreover, metallic nanoparticles such as aluminum and copper possess great thermal conductivities. However, in the current analysis, we consider the magneto-mixed convective flow of a hybrid Cu/Al_2_O_3_-water nanofluid about a radiated horizontal circular cylinder with thermal convective boundary conditions. A magnetic strength B_0_ is enforced in the trend normal to the fluid flow. Figure 1 demonstrates the schematic physical model and geometrical configuration of the considered investigation. Here *x* is measured about the cylinder surface and *y* is measured perpendicular to the surface.

Heat transfer within the hybrid nanofluid is analyzed by keeping constant temperature T_f_ as the cylinder surface, where T_f_ > T_∞_ (assisting flow) and T_f_ < T_∞_ (opposing flow) where T_∞_ is the ambient temperature. Velocity of the external flow is dignified by ${\mathop{u}\limits^{\frown {}}}_{e}(x)$. The flow under investigation is designated with the equations of^41,42^;

1 $$\frac{\partial \mathop{u}\limits^{\frown {}}}{\partial x}+\frac{\partial \mathop{v}\limits^{\frown {}}}{\partial y}=0,$$

2 $$\mathop{u}\limits^{\frown {}}\frac{\partial \mathop{u}\limits^{\frown {}}}{\partial x}+\mathop{v}\limits^{\frown {}}\frac{\partial \mathop{u}\limits^{\frown {}}}{\partial y}={\mathop{u}\limits^{\frown {}}}_{e}\frac{{\rm{d}}{\mathop{u}\limits^{\frown {}}}_{e}}{{\rm{d}}x}+\frac{{\mu }_{hna}}{{\rho }_{hna}}\frac{{\partial }^{2}\mathop{u}\limits^{\frown {}}}{\partial {y}^{2}}+\frac{{\sigma }_{hna}}{{\rho }_{hna}}({\mathop{u}\limits^{\frown {}}}_{e}-\mathop{u}\limits^{\frown {}})\pm \frac{1}{{\rho }_{hna}}{g}^{\ast }{(\rho \beta )}_{hna}(T-{T}_{\infty })\sin \left(\frac{x}{a}\right),$$

3 $$\mathop{u}\limits^{\frown {}}\frac{\partial T}{\partial x}+\mathop{v}\limits^{\frown {}}\frac{\partial T}{\partial y}={\alpha }_{hna}\frac{{\partial }^{2}T}{\partial {y}^{2}}-\frac{1}{{(\rho {C}_{p})}_{hna}}\frac{\partial {q}_{r}}{\partial y},$$

with the boundary conditions;

$$\mathop{u}\limits^{\frown {}}=\mathop{v}\limits^{\frown {}}=0,-{k}_{hna}\frac{\partial T}{\partial y}={h}_{f}({T}_{f}-T),\,{\rm{on}}\,y=0.$$

4 $$\mathop{u}\limits^{\frown {}}\to {\mathop{u}\limits^{\frown {}}}_{e}(x),T\to {T}_{\infty }\,{\rm{on}}\,y\to \infty .$$

where $\mathop{u}\limits^{\frown {}}$ and $\mathop{v}\limits^{\frown {}}$ are the velocity components along *x* and *y* axes, respectively, T is the temperature in the fluid phase. ${\rho }_{hna}$ stands for the density. ${\mu }_{hna}$ stands for viscosity. g* stands for gravitational acceleration. ${\beta }_{hna}$ stands for the hybird nanofluid volumetric thermal expansion coefficient. *σ*_*hna*_ stands for the electrical conductivity. ${\alpha }_{hna}={k}_{hna}/{(\rho {C}_{p})}_{hna}$ stands for the thermal diffusivity of the hybrid nanofluid. The velocity of the external flow ${\mathop{u}\limits^{\frown {}}}_{e}(x)$ is given by ${\mathop{u}\limits^{\frown {}}}_{e}(x)={U}_{\infty }\,\sin (x/a)$, where U_∞_ is the free stream velocity and *a* is the radius of the cylinder. The last term on R.H.S. of Eq. (2) exhibits the effects of the thermal buoyancy force, with positive and negative indication respecting to the buoyancy assisting and opposing flow, respectively. The radiative heat flux term is characterized by the Rosseland diffusion approximation is given by, see^43,44^;

5 $${q}_{r}=-\frac{4{\sigma }_{1}}{3{\beta }_{R}}\frac{\partial {T}^{4}}{\partial y}=-\frac{16{\sigma }_{1}{T}^{3}}{3{\beta }_{R}}\frac{\partial T}{\partial y},$$

with *σ*_1_ stands for the Stefan-Boltzmann constant. *β*_*R*_ stands for the Rosseland extinction coefficient. The term $16\sigma {T}^{3}/3{\beta }_{R}$ is specified as the radiative conductivity.

To gain the nonsimilar data, apply the following non-dimensional variables;

6 $$\begin{array}{c}\xi =x/a,\eta ={\mathrm{Re}}^{1/2}(y/a),u=\mathop{u}\limits^{\frown {}}/{U}_{\infty },v={\mathrm{Re}}^{1/2}(\mathop{v}\limits^{\frown {}}/{U}_{\infty }),{u}_{e}(\xi )={\mathop{u}\limits^{\frown {}}}_{e}(x)/{U}_{\infty },\\ \theta (\xi ,\eta )=(T-{T}_{\infty })/({T}_{f}-{T}_{\infty }).\end{array}$$

In the present investigation, the hypothetical relationships are characterized as follows:

7 $$\frac{\partial u}{\partial \xi }+\frac{\partial v}{\partial \eta }=0,$$

8 $$\begin{array}{c}u\frac{\partial u}{\partial \xi }+v\frac{\partial u}{\partial \eta }={u}_{e}\frac{{\rm{d}}{u}_{e}}{{\rm{d}}\xi }+\frac{{\rho }_{f}}{{\rho }_{hna}}\frac{{\mu }_{hna}}{{\mu }_{f}}\frac{{\partial }^{2}u}{\partial {\eta }^{2}}+\frac{{\rho }_{f}}{{\rho }_{hna}}\frac{{(\rho \beta )}_{hna}}{{(\rho \beta )}_{f}}\lambda \theta \,\sin \,\xi \\ +\frac{{\rho }_{f}}{{\rho }_{hna}}\frac{{\sigma }_{hna}}{{\sigma }_{f}}H{a}^{2}({u}_{e}-u),\end{array}$$

9 $$u\frac{\partial \theta }{\partial \xi }+v\frac{\partial \theta }{\partial \eta }=\frac{1}{\Pr }\frac{{\alpha }_{hna}}{{\alpha }_{f}}\frac{{\partial }^{2}\theta }{\partial {\eta }^{2}}+\frac{4Rd}{3\Pr }\frac{{(\rho {C}_{p})}_{f}}{{(\rho {C}_{p})}_{hna}}\{\theta {\prime} {[(H-1)\theta +1]}^{3}\}{\prime} .$$

The dimensionless boundaries are defined as:

$$u=v=0,\frac{{k}_{hna}}{{k}_{f}}\frac{\partial \theta }{\partial \eta }=-Bi[1-\theta ],\,{\rm{on}}\,\eta =0$$

10 $$u\to {u}_{e},\theta \to 0\,{\rm{as}}\,\eta \to \infty ,$$

where the following dimensionless variables are used

11 $$\begin{array}{c}\lambda =\frac{{g}^{\ast }{\beta }_{f}({T}_{f}-{T}_{\infty })a}{{U}_{\infty }^{2}}=\pm \frac{Gr}{{\mathrm{Re}}^{2}},Gr=\frac{g{\beta }_{f}({T}_{f}-{T}_{\infty }){a}^{3}}{{\upsilon }_{f}^{2}},\mathrm{Re}={U}_{\infty }a/{\upsilon }_{f},\Pr =\frac{{\upsilon }_{f}}{{\alpha }_{f}},\\ Rd=4{\sigma }_{1}{T}_{\infty }^{3}/{\beta }_{R}{k}_{f},H={T}_{w}/{T}_{\infty },Bi=\frac{{h}_{f}a}{{\mathrm{Re}}^{1/2}{k}_{f}},Ha={B}_{0}a{\left(\frac{{\sigma }_{f}}{\mathrm{Re}{\mu }_{f}}\right)}^{1/2},\end{array}$$

where λ stands for the mixed convection parameter. Gr stands for the Grashof number. Re stands for Reynolds number. Pr stands for the Prandtl number. Rd stands for the thermal radiation parameter. H is the surface temperature excess ratio. Bi stands for the Biot number. Ha stands for the Hartmann number.

Using the following hybrid nanofluid parameters;

$$\begin{array}{rcl}{\rho }_{hna} & = & {\phi }_{A{l}_{2}{O}_{3}}{\rho }_{A{l}_{2}{O}_{3}}+{\phi }_{Cu}{\rho }_{Cu}+(1-\phi ){\rho }_{f},{(\rho {C}_{p})}_{hna}={\phi }_{A{l}_{2}{O}_{3}}{(\rho {C}_{p})}_{A{l}_{2}{O}_{3}}+{\phi }_{Cu}{(\rho {C}_{p})}_{Cu}\\  &  & +(1-\phi ){(\rho {C}_{p})}_{f},{(\rho \beta )}_{hna}={\phi }_{A{l}_{2}{O}_{3}}{(\rho \beta )}_{A{l}_{2}{O}_{3}}+{\phi }_{Cu}{(\rho \beta )}_{Cu}\\  &  & +(1-\phi ){(\rho \beta )}_{f},{\alpha }_{hna}=\frac{{k}_{hna}}{{(\rho {C}_{p})}_{hna}},{\mu }_{hna}=\frac{{\mu }_{f}}{{(1-({\phi }_{A{l}_{2}{O}_{3}}+{\phi }_{Cu}))}^{2.5}},\end{array}$$

12 $$\begin{array}{rcl}\frac{{k}_{hna}}{{k}_{f}} & = & \left(\frac{({\phi }_{A{l}_{2}{O}_{3}}{k}_{A{l}_{2}{O}_{3}}+{\phi }_{Cu}{k}_{Cu})}{\phi }+2{k}_{f}+2({\phi }_{A{l}_{2}{O}_{3}}{k}_{A{l}_{2}{O}_{3}}+{\phi }_{Cu}{k}_{Cu})-2\phi {k}_{f}\right)\\  &  & \times {\left(\frac{({\phi }_{A{l}_{2}{O}_{3}}{k}_{A{l}_{2}{O}_{3}}+{\phi }_{Cu}{k}_{Cu})}{\phi }+2{k}_{f}-({\phi }_{A{l}_{2}{O}_{3}}{k}_{A{l}_{2}{O}_{3}}+{\phi }_{Cu}{k}_{Cu})+\phi {k}_{f}\right)}^{-1},\\ \frac{{\sigma }_{hna}}{{\sigma }_{f}} & = & 1+\frac{3\left(\frac{({\phi }_{A{l}_{2}{O}_{3}}{\sigma }_{A{l}_{2}{O}_{3}}+{\phi }_{Cu}{\sigma }_{Cu})}{{\sigma }_{f}}-({\phi }_{A{l}_{2}{O}_{3}}+{\phi }_{Cu})\right)}{\left(\frac{({\phi }_{A{l}_{2}{O}_{3}}{\sigma }_{A{l}_{2}{O}_{3}}+{\phi }_{Cu}{\sigma }_{Cu})}{\phi {\sigma }_{f}}+2\right)-\left(\frac{({\phi }_{A{l}_{2}{O}_{3}}{\sigma }_{A{l}_{2}{O}_{3}}+{\phi }_{Cu}{\sigma }_{Cu})}{{\sigma }_{f}}-({\phi }_{A{l}_{2}{O}_{3}}+{\phi }_{Cu})\right)},\end{array}$$

where $\phi ={\phi }_{A{l}_{2}{O}_{3}}+{\phi }_{Cu}$ represents the overall volume concentration. The thermal and physical properties of nanofluids have been considered in^45,46^ (see Table 1). Applying Eqs. (6–7) in Eqs. (1–4), one can obtain (see Nazar *et al*.^41^);

**Table 1:** Thermophysical properties of nanoparticles^45,46^.

Property	Water	Copper (Cu)	Alumina (Al_2_O_3_)
$\rho$(kg/m^3^)	997.1	8933	3970
${C}_{p}$(J/kg.K)	4179	385	765
$k$(W/m.K)	0.613	401	40
$\beta$(1/K)	$36.2\times {10}^{-5}$	$1.67\times {10}^{-5}$	0.85 × 10^−5^
$\sigma$ (μS/cm)	0.05	5.96 × 10^7^	1 × 10^−10^
$\mu$(m^2^ s^−1^)	6.25 × 10^−4^	—	—

$\psi =\xi f(\xi ,\eta )$, $\theta =\theta (\xi ,\eta )$ where ψ stands for the dimensionless stream function characterized in the regular trend as: $u=\partial \psi /\partial \eta$ and $v=-\partial \psi /\partial \xi$. On account of the aforementioned assumptions, the flow governing equations transform to the following form:

13 $${A}_{1}f\prime\prime\prime +ff{\prime\prime} -{f{\prime} }^{2}+\frac{\sin \,\xi \,\cos \,\xi }{\xi }+{A}_{2}\theta -{A}_{3}\left(f{\prime} -\,\frac{\sin \,\xi }{\xi }\right)=\xi \left(f{\prime} \frac{\partial f{\prime} }{\partial \xi }-f{\prime\prime} \frac{\partial f}{\partial \xi }\right),$$

14 $$\frac{1}{\Pr }\,\frac{{\alpha }_{hna}}{{\alpha }_{f}}\theta {\prime\prime} +f\theta {\prime} +{A}_{4}\{\theta {\prime} {[(H-1))\theta +1]}^{3}\}{\prime} =\xi \left(f{\prime} \frac{\partial \theta }{\partial \xi }-\theta {\prime} \frac{\partial f}{\partial \xi }\right),$$

15 $$f=f{\prime} =0,\frac{{k}_{hna}}{{k}_{f}}\theta {\prime} =-\,Bi[1-\theta ],{\rm{at}}\,\eta =0,f{\prime} \to \frac{\sin \,\xi }{\xi },\theta \to 0,{\rm{as}}\,\eta \to \infty .$$

where $${A}_{1}=\frac{{\rho }_{f}}{{\rho }_{hna}}\frac{1}{{(1-\phi )}^{2.5}}\,,\,{A}_{2}=\frac{{(\rho \beta )}_{hna}}{{\rho }_{hna}\,{\beta }_{f}}\,\lambda \frac{\sin \,\xi }{\xi }\,,\,{A}_{3}=\frac{{\sigma }_{hna}}{{\sigma }_{f}}\,\frac{{\rho }_{f}}{{\rho }_{hna}}H{a}^{2},\,{A}_{4}=\frac{{(\rho {C}_{p})}_{f}}{{(\rho {C}_{p})}_{hnf}}\frac{4Rd}{3\Pr }.$$

The quantities of engineering interest are as such, the drag coefficient;

16 $$\begin{array}{rcl}{C}_{f}(\xi ) & = & {\mathrm{Re}}^{1/2}\frac{{\tau }_{w}}{{\rho }_{f}{U}_{\infty }^{2}}={\mathrm{Re}}^{1/2}\frac{{\mu }_{hna}{(\partial \mathop{u}\limits^{\frown {}}/\partial y)}_{y=0}}{{\rho }_{f}{U}_{\infty }^{2}}\\  & = & \frac{\xi }{{(1-\phi )}^{2.5}}f{\prime\prime} (\xi ,0)\end{array}$$

and, local Nusselt number;

17 $$Nu(\xi )=-\left(\frac{{k}_{hna}}{{k}_{f}}+\frac{4{H}^{3}Rd}{3}\right)\theta {\prime} (\xi ,0),$$

## Hybrid Linearization– Chebyshev Spectral Method (HLCSM)

The solution of the nonlinear Partial Differential Equations (PDEs) (13)–(15) has a boundary layer at which the solution varies rapidly and away from the layer the solution various slowly and hence accurate and efficient computational techniques are needed for solving the considered problem^47–52^.

System (13)–(15) can be written as;

18 $$f{\prime} =g,$$

19 $${A}_{1}g{\prime\prime} +fg{\prime} -{g}^{2}+\frac{\sin \,\xi \,\cos \,\xi }{\xi }+{A}_{2}\theta -{A}_{3}\left(g-\frac{\sin \,\xi }{\xi }\right)=\xi \left(g\frac{\partial g}{\partial \xi }-g{\prime} \frac{\partial f}{\partial \xi }\right),$$

20 $${A}_{4}\theta {\prime\prime} +f\theta {\prime} +{A}_{5}\{\theta {\prime} {[(H-1))\theta +1]}^{3}\}{\prime} =\xi \left(g\frac{\partial \theta }{\partial \xi }-\theta {\prime} \frac{\partial f}{\partial \xi }\right),$$

Subject to the BCs:

21 $$f=0,g=0,\frac{{k}_{hna}}{{k}_{f}}\theta {\prime} =-\,Bi[1-\theta ],\,{\rm{at}}\,\eta =0,g\to \frac{\sin \,\xi }{\xi },\theta \to 0,\,{\rm{as}}\,\eta \to \infty $$

Newton’s linearization method combined with Gauss-Seidel relaxation technique (NLGS) is utilized to linearize and decouple the nonlinear PDEs (18)–(21) which are solved using Chebyshev spectral method (CSM)^47–52^. Applying NLGS on PDEs (18)–(21) results in

22 $$\begin{array}{c}{f{\prime} }_{n+1}={g}_{n},\\ {A}_{1}{g{\prime\prime} }_{n+1}+p{1}_{n}{g{\prime} }_{n+1}+p{2}_{n}{g}_{n+1}+p{3}_{n}=p{4}_{n}\frac{\partial {g}_{n+1}}{\partial \xi }+p{5}_{n}\frac{\partial {f}_{n+1}}{\partial \xi },\\ q{1}_{n}{\theta {\prime\prime} }_{n+1}+q{2}_{n}{\theta {\prime} }_{n+1}+q{3}_{n}{\theta }_{n+1}+q{4}_{n}=q{5}_{n}\frac{\partial {\theta }_{n+1}}{\partial \xi }+q{6}_{n}\frac{\partial {f}_{n+1}}{\partial \xi },\,n=0,1,2,\mathrm{...}.,\end{array}$$

with BCs

23 $$\begin{array}{rcl}{f}_{n+1}(\xi ,0) & = & 0,\,{g}_{n+1}(\xi ,0)=0,\,{g}_{n+1}(\xi ,{\eta }_{\infty })=\frac{\sin (\xi )}{\xi },\\ \frac{{k}_{hnf}}{{k}_{f}}{\theta {\prime} }_{n+1}(\xi ,0) & = & -Bi(1-{\theta }_{n+1}(\xi ,0)),\,{\theta }_{n+1}(\xi ,{\eta }_{\infty })=0,\,n=0,1,2,\mathrm{...}.\end{array},$$

The coefficients in (22) are defined by

24 $$\begin{array}{rcl}p{1}_{n} & = & \left({f}_{\bar{n}}+\xi \frac{\partial {f}_{\bar{n}}}{\partial \xi }\right),\,p{2}_{n}=\left(-2{g}_{n}-{A}_{3}-\xi \frac{\partial {g}_{n}}{\partial \xi }\right),p{4}_{n}=\xi {g}_{n},p{5}_{n}=0,\\ p{3}_{n} & = & \left({g}_{n}^{2}+{A}_{2}{\theta }_{n}+{A}_{3}\frac{\sin \,\xi }{\xi }+\frac{\sin \,\xi \,\cos \,\xi }{\xi }+\xi {g}_{n}\frac{\partial {g}_{n}}{\partial \xi }\right),\,q{1}_{n}=({A}_{4}+{A}_{5}{B}_{n}^{3}),\\ q{2}_{n} & = & \left(\xi \frac{\partial {f}_{\bar{n}}}{\partial \xi }+{f}_{\bar{n}}+2{A}_{5}(H-1){B}_{n}^{2}{\theta {\prime} }_{n}\right),\\ q{3}_{n} & = & {A}_{5}B(H-1)(3{\theta {\prime\prime} }_{n}{B}_{n}+2(H-1){({\theta {\prime} }_{n})}^{2})\\ q{4}_{n} & = & {A}_{5}(\,-\,3{\theta {\prime\prime} }_{n}{B}_{n}^{2}(H-1){\theta }_{n}+(H-1)(\,-\,{B}_{n}^{2}{({\theta {\prime} }_{n})}^{2}-2{({\theta {\prime} }_{n})}^{2}{B}_{n}(H-1){\theta }_{n})),\\  &  & q{5}_{n}=\xi {g}_{\bar{n}},q{6}_{n}=0\\ {B}_{n} & = & 1+(H-1){\theta }_{n},\end{array},$$

where $${f}_{n}={f}_{n}({\xi }_{j},{\eta }_{k})$$, $${g}_{n}={g}_{n}({\xi }_{j},{\eta }_{k})$$, $${\theta }_{n}={\theta }_{n}({\xi }_{j},{\eta }_{k})$$.

Equations (22)–(23) are a decoupled linear PDEs system where the terms subscripted by $n$ or $\bar{n}$ are known from the previous iteration or updated from the current iteration level, respectively, and the terms subscripted by $n+1$ are the current approximation. To solve the linearized system (22)–(23), we employed CSM in $\eta$-direction and the two-point implicit finite difference method^50^ in $\xi$-direction. The problem mesh grid-points (${\xi }_{j},{\eta }_{\kappa }$) are defined by:

25 $${\xi }_{j}=j{\Delta }_{\xi },\,{\eta }_{\kappa }=\frac{{\bar{\eta }}_{\infty }}{2}\left[1-\,\cos \,\frac{\kappa \pi }{{N}_{{\bar{\eta }}_{\infty }}}\right],j=0,1,\mathrm{...}.{N}_{\xi },\,\kappa =0,1,\mathrm{...}.{N}_{{\bar{\eta }}_{\infty }},$$

where ${\Delta }_{\xi }$ is the step-size in $\xi$-direction, ${\bar{\eta }}_{\infty }$ is the initial estimation of ${\eta }_{\infty }$, and $\,{N}_{\xi }\,,\,{N}_{{\bar{\eta }}_{\infty }}$ are the number of subintervals in $\xi$ and $\eta$-directions, respectively. The linear system (22)–(23) is transformed in the $\eta$-direction, and for each line ${\xi }_{j}$, into an algebraic system using the following linear differential transformation

26 $$\begin{array}{c}{{\bf{F}}}_{n+1,\,(j,\,\kappa )}^{(\mathop{m}\limits^{\frown {}})}={{\bf{D}}}^{\mathop{m}\limits^{\frown {}}}{{\bf{F}}}_{n+1,(j,\kappa )},\\ {{\bf{G}}}_{n+1,\,(j,\,\kappa )}^{(\mathop{m}\limits^{\frown {}})}={{\bf{D}}}^{\mathop{m}\limits^{\frown {}}}{{\bf{G}}}_{n+1,(j,\kappa )},\\ {{\boldsymbol{\Theta }}}_{n+1,\,(j,\,\kappa )}^{(\mathop{m}\limits^{\frown {}})}={{\bf{D}}}^{\mathop{m}\limits^{\frown {}}}{{\boldsymbol{\Theta }}}_{n+1,(j,\kappa )},\end{array}\},\,j=0,1,\mathrm{...}.{N}_{\xi },\,\kappa =0,1,\mathrm{...}.{N}_{{\bar{\eta }}_{\infty }},n=0,1,2,\mathrm{...}.,$$

where ${{\bf{D}}}^{\mathop{m}\limits^{\frown {}}}$, $\,\mathop{m}\limits^{\frown {}}=1,2\,,\,$ are the ${\mathop{m}\limits^{\frown {}}}^{th}$ derivatives Chebyshev differentiation matrices defined in^47–52^ and transformed into our entire domain $[0,\,{\bar{\eta }}_{\infty }]$, ${{\bf{F}}}_{n+1,(j,\kappa )}$, ${{\bf{G}}}_{n+1,(j,\kappa )}$ and ${{\boldsymbol{\Theta }}}_{n+1,(j,\kappa )}$ are the solution vectors ${[{f}_{n+1}({\xi }_{j},{\eta }_{\kappa })]}_{\kappa =0}^{{N}_{{\bar{\eta }}_{\infty }}}$, ${[{g}_{n+1}({\xi }_{j},{\eta }_{\kappa })]}_{\kappa =0}^{{N}_{{\bar{\eta }}_{\infty }}}$ and ${[{\theta }_{n+1}({\xi }_{j},{\eta }_{\kappa })]}_{\kappa =0}^{{N}_{{\bar{\eta }}_{\infty }}}$, respectively, while ${{\bf{F}}}_{n+1,\,(j,\,\kappa )}^{(\mathop{m}\limits^{\frown {}})}$, ${{\bf{G}}}_{n+1,\,(j,\,\kappa )}^{(\mathop{m}\limits^{\frown {}})}$ and ${{\boldsymbol{\Theta }}}_{n+1,\,(j,\,\kappa )}^{(\mathop{m}\limits^{\frown {}})}$ are the derivative vectors ${[{f}_{n+1}^{(\mathop{m}\limits^{\frown {}})}({\xi }_{j},{\eta }_{\kappa })]}_{\kappa =0}^{{N}_{{\bar{\eta }}_{\infty }}\,}$, ${[{g}_{n+1}^{(\mathop{m}\limits^{\frown {}})}({\xi }_{j},{\eta }_{\kappa })]}_{\kappa =0}^{{N}_{{\bar{\eta }}_{\infty }}\,}$ and ${[{\theta }_{n+1}^{(\mathop{m}\limits^{\frown {}})}({\xi }_{j},{\eta }_{\kappa })]}_{\kappa =0}^{{N}_{{\bar{\eta }}_{\infty }}\,}$, respectively. In $\xi$-direction, a two-point backward difference scheme^50^ similar to:

27 $${\frac{\partial \Pi }{\partial \xi }|}_{(j,\kappa )}=\frac{{\varPi }_{(j,\kappa )}-{\Pi }_{(j-1,\kappa )}}{{\Delta }_{\xi }},\,j=0,1,\mathrm{...}.{N}_{\xi },\,\kappa =0,1,\mathrm{...}.{N}_{{\bar{\eta }}_{\infty }}.$$

where $\Pi =g(\xi ,\eta )$ or $\theta (\xi ,\eta )$ is used to discretized first order derivatives with respect to $\xi$.

By applying CSM on (22)–(23), we have the following algebraic linear systems for each line ${\xi }_{j}$

28 $$\begin{array}{c}{[{{\bf{D}}}^{1}{{\bf{F}}}_{n+1}]}_{(j,\kappa )}={[{{\bf{G}}}_{n}]}_{(j,\kappa )}\\ {\left[{\boldsymbol{(}}{A}_{1}{{\bf{D}}}^{2}+{\boldsymbol{p}}{{\boldsymbol{1}}}_{n}{{\bf{D}}}^{1}{\boldsymbol{)}}{{\bf{G}}}_{n+1}+\left({\boldsymbol{p}}{{\boldsymbol{2}}}_{n}+\frac{{\boldsymbol{p}}{{\boldsymbol{4}}}_{n}}{{\Delta }_{\xi }}\right){\boldsymbol{.}}{{\bf{G}}}_{n+1}+{\boldsymbol{p}}{{\boldsymbol{3}}}_{n}\right]}_{(j,\kappa )}=\frac{{\boldsymbol{p}}{{\boldsymbol{4}}}_{n}}{{\Delta }_{\xi }}{\boldsymbol{.}}{{\bf{G}}}_{(j-1,\kappa )}\\ {\left[{\boldsymbol{(}}{\boldsymbol{q}}{{\boldsymbol{1}}}_{n}{{\rm{D}}}^{2}+{\boldsymbol{q}}{{\boldsymbol{2}}}_{n}{{\rm{D}}}^{1}{\boldsymbol{)}}{{\boldsymbol{\Theta }}}_{n+1}+\left({\boldsymbol{q}}{{\boldsymbol{3}}}_{n}+\frac{{\boldsymbol{q}}{{\boldsymbol{5}}}_{n}}{{\Delta }_{\xi }}\right){\boldsymbol{.}}{{\boldsymbol{\Theta }}}_{n+1}{\boldsymbol{+}}{\boldsymbol{q}}{{\boldsymbol{4}}}_{n}\right]}_{(j,\kappa )}=\frac{{\boldsymbol{q}}{{\boldsymbol{5}}}_{n}}{{\Delta }_{\xi }}{\boldsymbol{.}}{{\boldsymbol{\Theta }}}_{(j-1,\kappa )}\\ {{\bf{F}}}_{n+1,(j,0)}=0,\,{{\bf{G}}}_{n+1,(j,0)}=0,\,{{\bf{G}}}_{n+1,(j,{N}_{{\bar{\eta }}_{\infty }})}=\frac{\sin ({\xi }_{j})}{{\xi }_{j}},\\ \,\frac{{k}_{hna}}{{k}_{f}}{{\boldsymbol{(}}{{\bf{D}}}^{1}{\boldsymbol{\Theta }}{\boldsymbol{)}}}_{n+1,(j,0)}=-\,Bi(1-{{\boldsymbol{\Theta }}}_{n+1,(j,0)}),\,{{\boldsymbol{\Theta }}}_{n+1,(j,{N}_{{\bar{\eta }}_{\infty }})}=0.\end{array}\},$$

where the coefficients in (28) are the vector forms of the coefficients defined in (24). System (28) is solved iteratively at each line ${\xi }_{j}$, $\,j=0,1,\mathrm{...}.{N}_{\xi }\,$ and the iterative procedure is stopped at

29 $${E}_{j}=\,{\rm{\max }}({\Vert {{\bf{F}}}_{n+1,(j,\kappa )}-{{\bf{F}}}_{n,(j,\kappa )}\Vert }_{\infty },\Vert \,{{\boldsymbol{G}}}_{n+1,(j,\kappa )}-{{\boldsymbol{G}}}_{n,(j,\kappa )}\Vert ,\Vert \,{{\boldsymbol{\Theta }}}_{n+1,(j,\kappa )}-{{\boldsymbol{\Theta }}}_{n,(j,\kappa )}\Vert ) < {10}^{-5}.$$

To start the iterative solution for (28) the initial guesses are chosen satisfying the BCs such as follows:

30 $$\begin{array}{rcl}{f}_{0}(0,\eta ) & = & \frac{\sin \,\xi }{\xi }({e}^{-\eta }-1+\eta )\\ {g}_{0}(0,\eta ) & = & \frac{\sin \,\xi }{\xi }(1-{e}^{-\eta })\\ {\theta }_{0}(0,\eta ) & = & 1-{e}^{{k}_{f}Bi/{k}_{hnf}(\eta -{\bar{\eta }}_{\infty })}\end{array},$$

**Figure 2:**
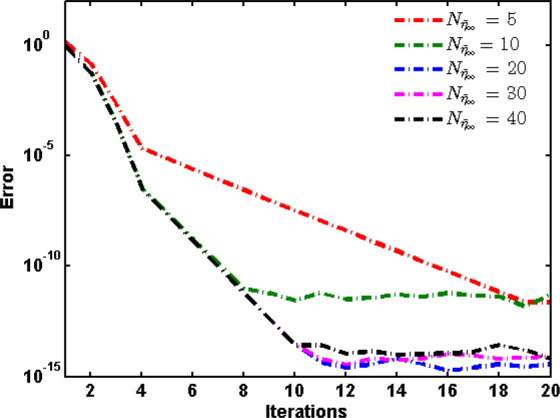
The maximum absolute error *E* versus the number of iterations for different numbers of collocation points ${N}_{{\bar{\eta }}_{\infty }}$.

Figure 2 shows the error $E=\,\max \,{\{{E}_{j}\}}_{j=0}^{{90}^{{\rm{o}}}}$ versus the number of iterations for different numbers of collocation points ${N}_{{\bar{\eta }}_{\infty }}$. It is clear that the algorithm convergence to the exact solution even at a low number of collocation points and the accuracy increases as the number of iterations increases.

**Figure 3:**
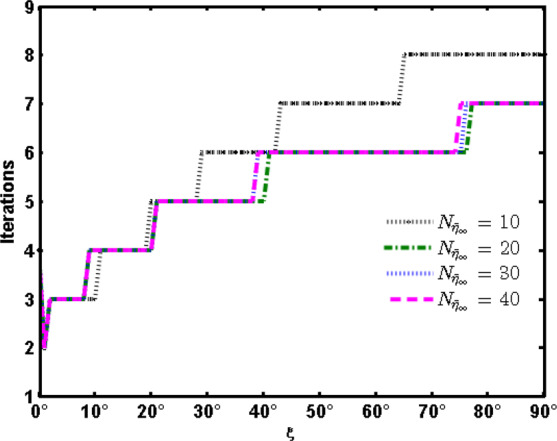
Required number of iterations with the variation of $\xi$ at different numbers of collocation points ${N}_{{\bar{\eta }}_{\infty }}$.

Figure 3 shows the required number of iterations to achieve a tolerance $E < {10}^{-5}$ with the variation of $\xi$ at different numbers of collocation points ${N}_{{\bar{\eta }}_{\infty }}$. It is clear that as $\xi$ increases, the number of iterations increases where the effect of the relaxed linearized right hand side terms increases. Moreover, at each line ${\xi }_{j}$ and for sufficient number of collocation points the desired accuracy is obtained and the number of iterations changes slightly with increasing the collocation points

**Figure 4:**
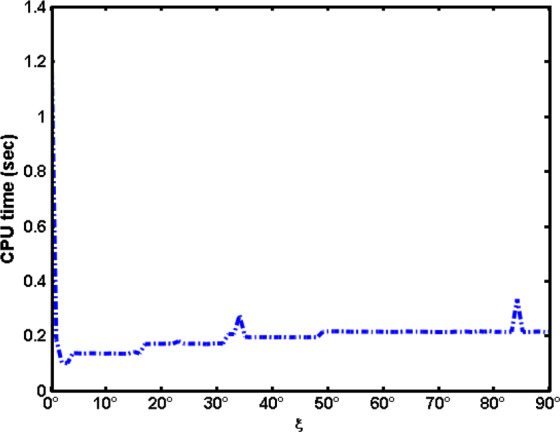
CPU computational time (sec) with variation of $\xi$.

Figure 4 shows the CPU computational time (sec) with variation of $\xi$. It is clear that the computational time at $\xi =0$ is dominant where the initial guesses are given by solution satisfying the BCs and given by (29) while for $\xi  > 0$, the initial guesses are the obtained solution at the previous step.

The accuracy of the above-mentioned computational technique was validated by direct comparisons with the numerical outcomes obtained previously by Merkin^41^ and Nazar *et al*.^42^ for several values of λ for a pure Newtonian fluid (*ϕ* = 0.0) at $Rd=Ha=0$ and $Bi\to \infty$ (in the absence of magnetic strength and thermal radiation across isothermal cylinder) at the lower stagnation point of the cylinder, i.e., $\xi \approx 0$. For small values of |λ| the forced convection impacts predominate, whilst for great |λ|, it is the free convection which is remarkable, so that values of λ of O(1), where both impacts are comparable and most interest. Nazar *et al*.^41^ explored that the local Nusselt number boost with raising the buoyancy forces for assisting/opposing flows. It is manifested that the impact of buoyancy forces on the forced convective becomes considerable for λ < −1.75 and λ > 5, for opposing and assisting flows, respectively. Table 2 presents a comparison of the numerical solution established by Merkin^41^ and Nazar *et al*.^42^ approaches and the HLCSM solution. It is found from this table that excellent agreement between the outcomes exists. This convenient comparison supplies confidence in the numerical data to be carried out in the next segment.

**Table 2:** Comparison of $-\theta {\prime} (0)$ for different values of λ for Ha = ϕ= Rd = 0, Bi → ∞ and *Pr* = 1.0.

λ	Merkin^41^	Nazar *et al*.^42^	Present Results
−1.75	0.4199	0.4205	0.4198
−1.5	0.4576	0.4601	0.4573
−1.0	0.5067	0.5080	0.5067
−0.5	0.5420	0.5430	0.5421
0.0	0.5705	0.5710	0.5705
0.5	0.5943	0.5949	0.5945
0.88	0.6096	0.6112	0.6108
0.89	0.6110	0.6116	0.6112
1.0	0.6158	0.6160	0.6154
2.0	0.6497	0.6518	0.6515
5.0	0.7315	0.7320	0.7315

## Results and Discussion

**Figure 5:**
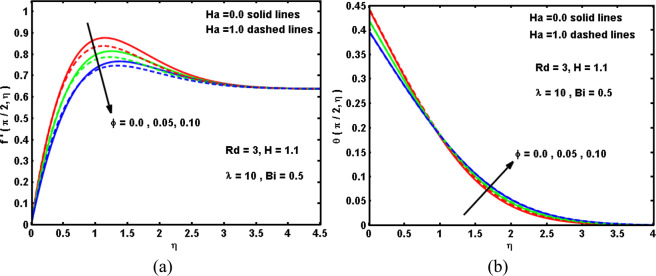
Impacts of Ha and ϕ on (**a**) velocity, and (**b**) temperature curves.

**Figure 6:**
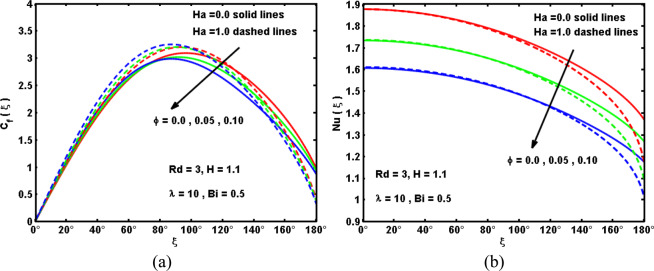
Impacts of Ha and ϕ on (**a**) drag coefficient, and (**b**) Nusselt number.

**Figure 7:**
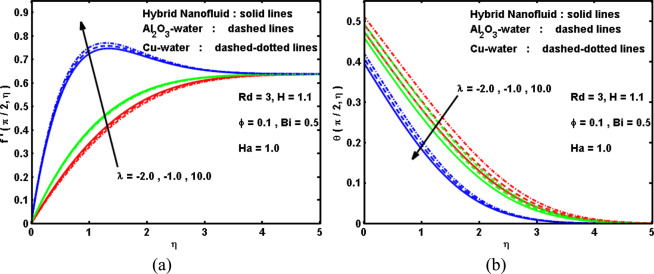
Impact of λ on (**a**) velocity, and (**b**) temperature curves.

**Figure 8:**
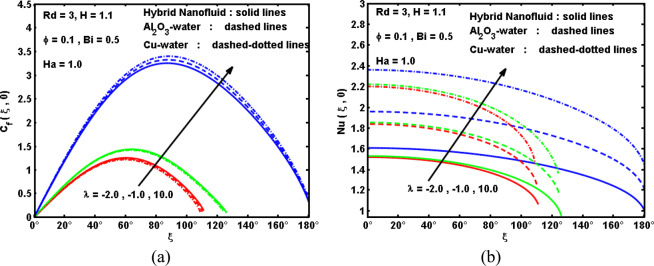
Impact of λ on (**a**) drag coefficient, and (**b**) Nusselt number.

**Figure 9:**
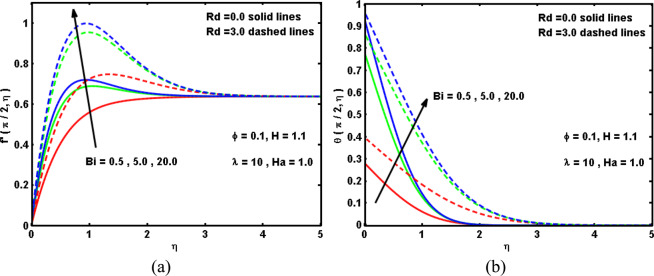
Impacts of Bi and Rd on (**a**) velocity, and (**b**) temperature curves.

**Figure 10:**
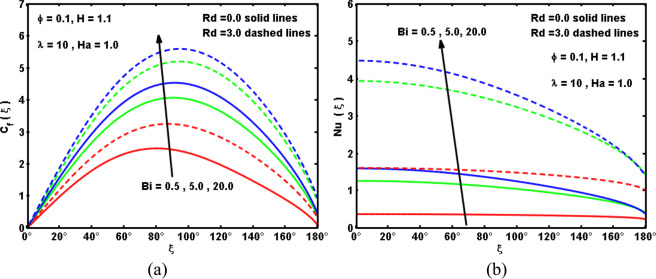
Impacts of Bi and Rd on (**a**) drag coefficient, and (**b**) Nusselt number.

In this segment, we explore the physical interpretations of the influence pertinent parameters on velocity $f{\prime} (\xi ,\eta )$, temperature $\theta (\xi ,\eta )$, drag coefficient C_f_(ξ) and local Nusselt number Nu(ξ). Plots are outlined to elucidate the outcomes through Figs. 5–10. Figure 5(a,b) exhibit the influence of the Hartmann number Ha and solid volume fraction of hybrid nanofluid ϕ on the hybrid nanofluid velocity $f{\prime} (\xi ,\eta )$ and temperature behaviour $\theta (\xi ,\eta )$. It is demonstrated that the boost in ϕ leads to inhibit the velocity behavior and to promote the hybrid nanofluids temperature far the surface because greater values of ϕ coincide to grow the hybrid nanofluid’s thermal conductivity (see Table 1) which prompts the heat dispersal consequently the heat rashly spread within the cylinder surface. Also, increasing the values of Hartmann number Ha corresponds to enhancing its strength, which in turn boosts the Lorentz force and decreases velocity while increases the temperature profile. Figure 6(a,b) are enumerated to record the diversity in the drag coefficient C_f_(ξ) and Nusselt number Nu(ξ) for numerous values of Ha and ϕ. It is interesting to note that the C_f_(ξ) elevates sufficiently by strengthening the Hartmann number Ha. This occurs because great values of Ha are answerable to enlarge the Lorentz force within boundary-layer region which opposes the flow in the reverse bearing, and based on that the Nusselt number declines. Moreover, It is portrayed that each of C_f_(ξ) and Nu(ξ) reduce by upsurge ϕ. Physically, the larger ϕ creates a low-energy convey across the flow near the surface concerned with the unequal motion of the nanoparticles, and hence make a massive reduction in the drag coefficient and heat transport.

Figure 7(a,b) interpret the effectiveness of mixed convection parameter λ on the velocity $f{\prime} (\xi ,\eta )$ and temperature $\theta (\xi ,\eta )$ curves with different forms of nanofluids (Cu, Al_2_O_3_, and hybrid). It can be noted that growing in the mixed parameter λ leads to raise the velocity curves due to convenient buoyancy impact and thus decline the temperature curves, and thereby thins the thermal boundary-layer. It is also remarked that the characteristic crests in the velocity curves move rapidly across the cylinder surface as λ boosts. Meanwhile, notice manifests that hybrid nanofluids have elevated the curves of the velocity and temperature. Also, velocity curves for the status of Cu-water is predominant as compare to Al_2_O_3_-water and Cu-Al_2_O_3_-water, and also, the temperature is high for the case of Cu-water as compare to Al_2_O_3_-water and Cu-Al_2_O_3_-water. Therefore, it can be said that hybrid technology may advantageous to improve the physical properties of the fluid. For this realization, it can be diminished the cost impacts. Figure 8(a,b) are sketched to record the variation in the drag coefficient C_f_(ξ) and Nusselt number Nu(ξ) for various values of λ for three kinds of nanofluids. As demonstrated above, an augmentation in the λ produces increment the buoyancy impact in a combined convection flow which causes an acceleration of the hybrid nanofluid flow about the cylinder surface. This yields an evolution in the drag coefficient as displayed in Fig. 7(a). Furthermore, the acceleration within the surface with increasing λ causes a decline in the thermal boundary layer thickness. This results in evolution in the local Nusselt number as appeared in Fig. 8(a,b). Furthermore, it is indicated that both C_f_(ξ) and Nu(ξ) have larger magnitude for Cu-water followed by Al_2_O_3_-water and hybrid nanofluid. As expected, the Cu-water nanofluid is the strongest intensification than other nanofluids, while the Al2O3-water nanofluid experiences the weakest intensification. The reason is that the thermal conductivity of Copper is greater than hybrid nanoparticles and Alumina nanoparticles. This means that the greats of heat transfer rate can be gained by adding Cu compared to other nanoparticles. It is also noticed from these Fig. 8(a,b), Also, for a given value of mixed convection parameter λ, the Nusselt number is visualized to decline with boosting the distance ξ from the stagnation point. Furthermore, it can be seen that, as expected, the boundary-layer separates from the cylinder for some minus values of λ (reversing flow) and also for some plus values of λ (assisting flow). Reversing flow convoys the separation point near the lower stagnation point and for appropriately great minus values of λ or appropriately robust reversing flow, there will be no boundary-layer on the cylinder. Boosting λ retards the separation and that separation can be totally restrained in the range 0 ≤ ξ ≤ 180° for sufficiently great values of λ (>0). However, the negative values of λ yield no separation, whilst the plus values produce that the boundary-layer keeps on the cylinder and begins to separate merely before the greater stagnation point (ξ = 180°) where values of the the drag coefficient C_f_(ξ) and Nusselt number Nu(ξ) become negative.

The impacts of Biot number Bi and radiation parameter *Rd* on the velocity and temperature curves are portrayed in Fig. 9(a,b). It is reported that both the velocity and temperature curves boost with the increment in the Biot number *Bi*. Physically, the convective heating boosts with growing the *Bi*, i.e., greater *Bi* demonstrate the isothermal surface, as portrayed in Fig. 9(b). In a vision of this demonstration, greater Bi supplies huge convective surface which in turn produces further warmth to the surface and thus the temperature variation between the nanofluid and the surface strengthens. On the other side, both the velocity and nanofluid temperature raise in the presence of radiation parameter *Rd*. These conducts inspire a considerable evolution in the thermal boundary layer thickness as *Rd* boosts. This outcome was predictable based on the formula of radiation phenomenon that Rd magnifies as thermal conductivity of the base fluid drops which causes magnify in the radiative heat rate which transmitted to the hybrid nanofluid and then the temperature declines. Finally, Fig. 10(a,b) plot the difference in the drag coefficient and the Nusselt number versus the circumferential position $\xi$ for several values of Bi and *Rd*. As above-mentioned, it is found that an evolution in *Rd* implies a prominent augmentation in the drag coefficient and Nusselt number. This harmonizes with the physical conduct that the heat transport becomes larger with the radiation impact, and hence the drag coefficient upsurges. Moreover, It is reported that an increasing in *Bi* causes an enhancement in the drag coefficient and Nusselt number. The cause for this conduct is that as *Bi* boosts, the hybrid nanofluid coldish on surface is convectively heated and thus, the velocity elevates, which in turn elevates in the drag coefficient and Nusselt number.

## Conclusions

Current investigation dedicated to analyze the magneto-mixed convective flow of Cu-Al_2_O_3_ nanofluid around a radiated circular cylinder with a convective surface. Significant outcomes are addressed below;

- The velocity curves enhance for assisting flow of hybrid nanofluids while it shows opposite behavior with large value of solid volume fraction.
- Biot number and radiation parameter accelerate the hybrid nanofluid motion while Hartmann number decelerates it.
- Temperature of hybrid nanofluid rises for assisting flow with radiation parameter, Biot and Hartmann numbers and but the contrary trend is observed for assisting flow.
- Drag coefficient enhances for assisting flow with radiation parameter, Biot and Hartmann numbers but the contrary trend is noted for solid volume fraction.
- Nusselt number occurs faster with Biot number and radiation parameter and slows down with Hartmann number and solid volume fraction.
- Both skin friction and Nusselt number have greater magnitude for Cu-water followed by hybrid nanofluid and Al_2_O_3_-water.
