# Author Correction: Magneto-Hybrid Nanofluids Flow via Mixed Convection past a Radiative Circular Cylinder

**DOI:** 10.1038/s41598-020-72921-8

**Published:** 2020-09-24

**Authors:** E. R. EL-Zahar, A. M. Rashad, W. Saad, L. F. Seddek

**Affiliations:** 1 Department of Mathematics, College of Science and Humanities in Al-Kharj, Prince Sattam Binin Abdulaziz University, Al-Kharj, 11942 Saudi Arabia; 2 Department of Basic Engineering Science, Faculty of Engineering, Menoufia University, Shebin El-Kom, 32511 Egypt; 3 Department of Mathematics, Aswan University, Faculty of Science, Aswan, 81528 Egypt; 4 Department of Engineering Mathematics and Physics, Faculty of Engineering, Zagazig University, Zagazig, 44519 Egypt

Correction to: *Scientific Reports*, 10.1038/s41598-020-66918-6 published online 26 June 2020

The original version of this Article contained an error in Affiliation 3, which was incorrectly given as ‘Departments of Mathematics, Aswan University, Faculty of Science, Aswan, 81528, Egypt’. The correct affiliation is listed below:

Department of Mathematics, Aswan University, Faculty of Science, Aswan, 81528, Egypt

In addition, the original version of this Article contained an error in Equation 11.

$$ H = T_{w} /T_{\infty } $$

now reads:

$$ H = T_{f} /T_{\infty } $$

Finally, this Article contained typographical errors in the Acknowledgments section.

“The authors would like to thank the referees for their valuable comments and suggestions which helped to improve the manuscript. Moreover, the authors would like to thank Prince Sattam bin Abdulaziz University, Deanship of Scientific Research at Prince Sattam bin Abdulaziz University for their continuous support and encouragement for this research project No. 2020/01/16595.”

now reads:

“The authors would like to thank the referees for their valuable comments and suggestions, which helped to improve the manuscript. Moreover, the authors would like to acknowledge that: This project was supported by the Deanship of Scientific Research at Prince Sattam bin Abdulaziz University under the research project No: 2020/01/16595.”

These errors have now been corrected in the HTML and PDF versions of the Article.

